# Evidence of a linkage between *matrilin-1 *gene (*MATN1*) and idiopathic scoliosis

**DOI:** 10.1186/1748-7161-1-21

**Published:** 2006-12-18

**Authors:** Lucio Montanaro, Patrizio Parisini, Tiziana Greggi, Mario Di Silvestre, Davide Campoccia, Simona Rizzi, Carla Renata Arciola

**Affiliations:** 1 Research Unit on Implant Infections, Molecular Pathology Section, Rizzoli Orthopaedic Institute, Bologna, Italy; 2 Experimental Pathology Department, University of Bologna, Italy; 3 Spine Surgical Division, Rizzoli Orthopaedic Institute, Bologna, Italy

## Abstract

**Background:**

In a previous study, a number of genes, associated with spine musculoskeletal deformity phenotypes in mouse and in synteny between mouse and man, were identified as candidate genes for IS. Among these genes, *MATN*1, which carries a polymorphic microsatellite marker within its sequence, was selected for a linkage analysis. *MATN1 *is localised at 1p35 and is mainly expressed in cartilage. The objective of this study was to assess a linkage disequilibrium between the *matrilin-1 *(*MATN*1) gene and the idiopathic scoliosis (IS).

**Methods:**

The genetic study was conducted on a population of 81 trios, each consistent of a daughter/son affected by idiopathic scoliosis (IS) and both parents. In all trios components, the region of *MATN1 *gene containing the microsatellite marker was amplified by a polymerase chain reaction. The amplicons were analysed by a DNA sequencer-genotyper. The statistical linkage analysis was performed using the extended transmission/disequilibrium test.

**Results:**

Three microsatellite polymorphisms, respectively consisting of 103 bp, 101 bp and 99 bp, were identified. ETDT evidenced a significant preferential transmission for the 103 bp allele (Chi-square = 5.058, df = 1, P = 0.024)

**Conclusion:**

The results suggest that the familial idiopathic scoliosis is associated to the *MATN1 *gene.

## Background

Adolescent idiopathic scoliosis (IS) is the most common spine deformity arising during childhood, but the etiology of IS remains unknown. A large proportion (75%) of structural scoliosis is clinically classified as idiopathic [1]. Idiopathic scoliosis often appears in several members of the same family, this strongly suggesting a genetic transmission. Clinical studies indicate that approximately 1:4 of the total scoliosis cases and 1:3 of idiopathic scoliosis cases are familial [2]. Also studies on twins showing that concordance of monozygotic twins is greater than that of dizygotic twins [3,4] suggest a genetic basis for the IS. Nevertheless, in addition to genetic traits, a number of other environment-related factors have been described [5] that variously contribute to the final expression/modulation of the phenotype in each single individual case.

A series of candidate genes, including *FBN*1, *COL1A*1, *COL1A*2, *COL2A1 *and *elastin *genes, have already been examined by linkage studies, with negative results [6,7], and, at present, the particular mode of inheritance of the idiopathic scoliosis still remains unclear. There are conflicting data in the existing literature. Some reports show that the disorder has many of the characteristics of a complex trait, indicating the presence of a multifactorial inheritance pattern, while other studies indicate a major autosomal dominant gene effect [8-10]. Even more, not all the linkage studies, which demonstrate that the inheritance pattern of idiopathic scoliosis is based on a major autosomal dominant gene effect, did identify a unique locus responsible for idiopathic scoliosis. A linkage with idiopathic scoliosis has been found at locus 17p11 in a three generation Italian family [10] and at locus 19p13.3 in a Chinese family [11]. Therefore, it is possible that idiopathic scoliosis is caused by alterations in different genes.

This study aimed at investigating the loci responsible for susceptibility to idiopathic scoliosis in all the population and not only in single families. For this reason, we chose to perform an association study on parent-offspring trios.

We selected the gene encoding for the protein Matrilin-1 (*MATN1 *gene) as a candidate gene for human idiopathic scoliosis, by reviewing mouse mutations with phenotypes affecting the musculoskeletal system and considering only those which presented synteny conservation between mouse and man [12,13]. Besides, the *MATN1 *gene is localised on chromosome 1p35 and has the advantage to contain within its sequence a microsatellite marker, which allows to follow its segregation in a population of trios.

*MATN1 *gene is mainly expressed in cartilage. Its proteic product, also known with the alternative name of cartilage matrix protein, is an extracellular matrix structural constituent, which is associated with cartilage proteoglycans as well as being a component of both collagen-dependent and collagen-independent fibrils [14].

Here, *MATN1 *has been studied as candidate gene for IS by an intragenic microsatellite marker localised in the 8th exon of the gene. The microsatellites are good markers because are very polymorphic and allow to follow the segregation of the candidate genes, which contain them, in family groups.

## Materials and methods

### Patient Selection

The object of the study consists of 81 trios, composed of a child affected by IS, his father and his mother. The probands had been operated in the previous years by the Rachis Surgery Division of the Rizzoli Orthopaedics Institutes and had undergone periodical check-up. Informed consent was obtained from all study participants for taking a sample of blood, which was necessary for the following Genotype Analysis.

The criteria adopted for the clinical diagnosis of scoliosis required a spinal curvature in the sagittal plane. Three different classes of scoliosis severity were then identified based on anteroposterior spinal radiograph curvature by the Cobb method with pedicle rotation (5–15° mild, 15–40° medium, >40° severe scoliosis). Starting the scale from a low degree of Cobb offered the possibility to keep track of even very slight levels of scoliosis in the parental generation of the probands. It has to be underlined that the presence of disease condition of the parents is not taken in consideration in the genetic test and, thus, is not influential on the results of the analysis, which uniquely examines the disequilibrium of inherited marker traits.

### DNA Extraction from Whole Blood

Genomic DNA was extracted from 5-ml blood samples with the "salting out" method as earlier described in Montanaro *et al*. (2002) [15]. The obtained DNA was prepared at a final concentration of 500 ng/μL and stored at -20°C.

### PCR of *MATN1 *region containing the microsatellite marker

The PCR reaction was carried out utilizing a Cy5-labeled forward primer. The sequence of the forward primer (positions 2135–2145 of *MATN1*, GenBank accession number NM_002379) was 5'-TAT GTG TGC GTG TGT GTA TG-3', while the sequence of the reverse primer (positions 2216–2235) was 5'-CTC TCA CAC TCA CAT TCT GG-3'.

All amplification reactions were carried out in the UNO II Thermocycler BIOMETRA in a volume of 25 μL containing 0.5 U of Taq DNA polymerase, 500 ng of extracted DNA, 400 mM each of dATP, dCTP, dGTP and dTTP, reaction buffer (10 mM Tris HCl, pH 9.0; 50 mM KCl; 0.1% Triton X-100) and 1.5 mM MgCl2. The thermal step program consisted of initial denaturation at 94°C for 5 minutes, 40 cycles of denaturation at 94°C for 30 seconds, annealing at 56°C for 30 seconds, extension at 72°C for 30 seconds, followed by a final extension at 72°C for 1 minute. After amplification, 7 μL of product were examined by electrophoresis on a 2.5% agarose gel. The Marker V (Roche) was used a molecular weight reference.

### Fragments analysis by CEQ8000 Genetic Analysis System

The Cy5-labeled PCR products, derived from all the components of the 81 trios, were analysed by the CEQ8000 Genetic Analysis System (Beckman Coulter) to determine the fragments length of the DNA samples and, thus, the genotype for the analysed microsatellite marker. The DNA samples were automatically denatured at 95°C for 2 minutes and then separated by capillary electrophoresis. Detection was by laser-induced fluorescence in four spectral channels.

The amplification products were first diluted 1:100 with SLS solution (Sample Loading Solution) containing deionised formamide. The utilised Size Marker was the Size Standard 400, Beckman Coulter, containing fragments labelled with the D1 Beckman label and ranging in size from 60 to 420 nucleotides. A Mix SLS solution was prepared with 40 μL of SLS and 0.5 μL of Size Standard for each sample, according to the manufacturer's protocol. 0.25 μL of each diluted sample was loaded in the microplate with 40.5 μL of the Mix SLS solution. The electrophoresis was performed at 6 kV and 50°C for 37 minutes. The information about the genotype of the microsatellite marker of each component of the trios was provided as a peak profile after the fragments analysis.

### Fragments analysis by Genomix LR-SC

The amplified fragments were also analysed by the genotyper Genomix LR-SC for a counter-check of the exactness of the fragments' length determined by CEQ8000. The PCR reactions to obtain amplified fragments were performed as per CEQ8000 analysis, but the labelling was carried out using a fluorescein-labelled forward primer. These fluorescein-labelled amplified fragments were diluted 1:10 with a loading dye containing 50% formamide, denatured at 95°C for 5 minutes and subsequently analysed by the Genomix LR-SC on a 6% polyacrylamide denaturing gel. The electrophoresis was performed at the following conditions: 125 W, 2750 V and 50°C for 75 minutes. The software Clarity SC calculated the length of the fragments on acquired images of the gel.

### Statistical analysis

The Extended Transmission Disequilibrium Test (ETDT) was employed to search for linkage disequilibrium [16-18]. The TDT statistic looks at the parents heterozygous for a specific marker allele. Then, it compares how often a specific marker allele is transmitted to the affected offspring from such heterozygous parents. If there is no linkage, then the considered allele would be transmitted 50% of the time as well as the other alternative allele. Vice versa, if there are linkage and association, the considered allele would most likely be on the chromosome with the disease allele, and they would be inherited together more than 50% of the time.

## Results of analysis of the fragments

**Figure 1:**
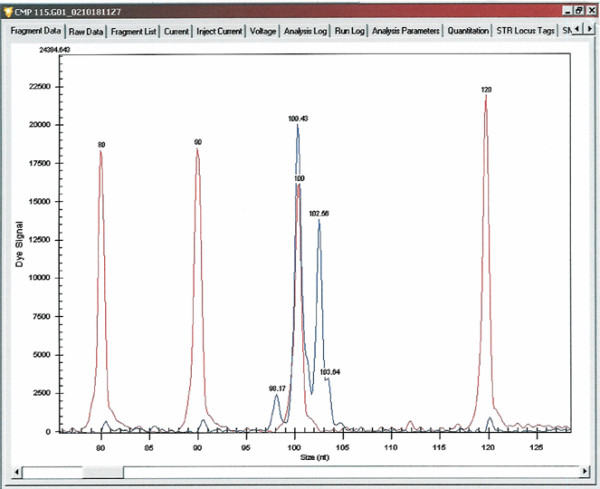
Detection of the genotype heterozygous 101 bp–103 bp for the microsatellite marker internal to *MATN1 *gene by the fragment analysis performed with the sequencer/genotyper CEQ8000. The red peaks are those of Size Standard, while the blue peaks correspond to the Cy5-labeled amplified fragments. The 100.43 nt-peak corresponds to the 101 bp allele and the102.56 nt-peak corresponds to the 103 bp allele.

**Figure 2:**
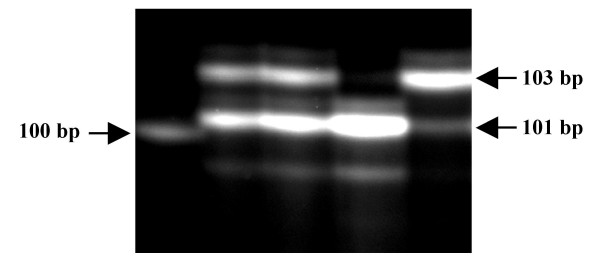
Detection of the genotype relative to the microsatellite internal to *MATN1 *gene through the analysis realized with Genomix LR-SC. 1) 100 bp band of the Size Standard; 2) and 3) Heterozygous sample 101 bp–103 bp; 4) Homozygous sample 101 bp–101 bp; 5) Homozygous sample 103 bp–103 bp.

The genotype analysis of microsatellite marker region of each component of the trios by CEQ8000 Genetic Analysis System (Beckman Coulter) provides a profile of three peaks (Figure 1). The analysis by the Genomix LR-SC genotyper always confirmed the results obtained with the CEQ8000. An example of the detection of the genotypes related to the microsatellite marker on *MATN1 *gene is shown in Figure 2. In the analysis with the Genomix LR-SC, the different genotypes are revealed by the bands appearing on the gel. The selected microsatellite marker showed a three variants-polymorphism consisting of 103 bp, 101 bp and 99 bp, which were observed with a frequency of 65.7%, 31.6% and 2.6%, respectively.

## Results of ETDT

The ETDT test was performed on all the 81 families of the study, of which 50 with the parents heterozygous for a specific marker allele (informative families). It was decided to limit the ETDT to the trios who presented the most frequent allelic variants i.e. 101 bp and 103 bp alleles, because the frequency of 99 bp allele in the population of parents of the affected probands (2.6%) was too low to provide any information about the model of transmission to affected offspring. It should have been necessary to screen a too large number of trios to obtain a number of parents sufficiently high to perform a statistical study on it.

We have looked for a particular allelic variant to be transmitted to offspring with a frequency higher than the 50%. It has been counted how many times each single allele was transmitted from the heterozygous parent to the affected proband and how many times it was not transmitted. The results of the ETDT (extended transmission disequilibrium test) are shown in Table 1. The ETDT conducted on all the 81 trios of the study rendered a chi-squared score for allele-wise analysis of 6.355804, which with 2 degrees of freedom (d.f.) was significant (p = 0.041757). It can be noticed that there is an allele that was more frequently transmitted (T) than not transmitted (NT) from parents to affected probands (T/NT = 45/26): this is allele 103 bp (transmission rate: 63%). On the contrary the allele 101 bp and the allele 99 bp were preferentially not transmitted, exhibiting a transmission rate of 25% and of 39%, respectively.

**Table 1:** Transmission of alleles of *MATN1 *gene in the 81 families of the study and ETDT results

**Allele**	**T**	**NT**	**T.R.**	**χ^2^**	**p**	**Chi-square statistics**
**99 bp**	2	6	25%			χ^2 ^for "allele-wise" ETDT = 6.355804, 2 gl, p = 0.041757
**101 bp**	28	43	39%	3.169	0.0751	χ^2 ^for "genotype-wise" ETDT = 11.007881, 3 gl, p = 0.011743
**103 bp**	45	26	63%	5.0585	0.0242	χ^2 ^for goodness-of-fit "allele-wise" model = 4.65207, 1 gl, p = 0.03106

T = number of transmitted alleles; NT = number of non-transmitted alleles; T.R.: transmission rate (%) = T/(T+NT) × 100.

ETDT is calculated as χ^2 ^= (T - NT)^2^/(T + NT), 1 d.f. for each allele and consequently a logistic regression is performed outputting chi-squared statistics with 2 d.f. for allele-wise analysis and 3 d.f. for genotype-wise analysis.

The transmission data and the statistical significance of results, analysed by the Extended Transmission Disequilibrium Test (ETDT), are illustrated in Table 1. Based on the results obtained in the family group analysed in this study, the ETDT test reveals a possible linkage between the allele 103 bp and idiopathic scoliosis.

## Discussion

The results obtained from the analysis of transmissions of the allelic variants of the microsatellite marker internal to *MATN1 *gene, in a population of trios composed of children affected by IS and their parents, show the presence of a linkage between the 103 bp allele and IS. The TDT test has the disadvantage that it can detect linkage between the marker locus and the disease locus only if association (due to linkage disequilibrium) is present, but since in the present study the TDT rendered a positive result in favour of the presence of linkage we can affirm that also an association must exist between the allelic variant 103 bp of *MATN1 *gene and susceptibility to IS. It is actually more correct to talk about susceptibility to scoliosis because the genetic expression of IS may be dependent on multiple factors and genetic interactions [5,19,20].

The particular mode of inheritance of IS is unclear. Some reports show that the disorder has many of the characteristics of a complex trait, indicating the presence of a multifactorial inheritance pattern [21,22], while other studies have produced evidence of a major autosomal dominant gene effect [8-10]. However, the different linkage studies, which demonstrate that the inheritance pattern of IS is based on a major autosomal dominant gene effect, do not find a unique locus responsible for IS. The group of Wise in a genome-wide linkage survey identified a limited number of genetic loci predisposing to IS: maximum evidence of allele-sharing in one family was detected for three loci on chromosome 6p, distal 10q, and 18q [23]. Another study found a linkage with IS at locus 17p11 in a three generation IS Italian family 9 and another linkage with IS was found at locus 19p13.3 in a Chinese family [11]. A recent study reports that also an X-linked susceptibility locus seems be involved in the expression of familial IS [24]. The complex of the information gained from the studies of linkage suggests many possible interpretations of genetics of IS. It is possible that this disease may be caused by defects in multiple genes or that many kinds of IS exist, each of these due to the effect of a different major gene.

All these linkage studies have the limit to find loci linked to IS in particular families. Our study, which was performed on a large number of trios and not on a single large pedigree, has the advantage that it can simultaneously reveal association and linkage between a marker locus and a disease locus, if present, reminding that "association" is a relation between alleles and that "linkage" is a relation between loci. In other words, this study conducted on parent-offspring trios was aimed at finding the allele of a marker linked to a locus responsible of susceptibility to IS in all the population and not just within specific families.

We did find a linkage and an association with the allele marker 103 bp on *MATN1 *gene, that maps on locus 1p35. Our study design is rather original in the field of investigation of genetic bases of IS. In the literature there is only one study performed on a sample of parent-offspring trios, searching for a linkage with IS utilising the approach of the candidate gene [25]. These authors conducted a linkage analysis of a polymorphism of the number of tandem repeats in the *aggrecan *gene, but they did not find any linkage.

Although the association that we observe between *MATN1 *gene and IS is not complete, this result is relevant because it brings to the identification of one of the loci that may contribute to susceptibility to IS in a large population. If the hypothesis of the multifactorial inheritance model is the right one, the association between the IS character and the 103 bp-allelic variant is not complete because of the scoliosis phenotype can be the result of the contribute of several genes. The detection of a linkage disequilibrium between the allelic variant 103 bp of the microsatellite in *MATN1 *gene in a study performed on trios means that it has been localized an allelic variant that, in an ancestor common to all affected people, should have been associated with the mutation causative of the disease. Even more, this allelic variant should have been localised at such a little distance that it did not undergo any recombination event during the time.

The TDT is not able to distinguish between associations caused by linkage disequilibrium and those where the marker is itself a factor of susceptibility. To find the mutation causative to expression of IS it would be necessary to analyse the entire sequence of *MATN1 *gene in the affected probands who present the 103 bp allele.

Anyway, as we are dealing with an intragenic microsatellite marker localized in 3' untranslated region (3'UTR) of the gene, which codifies for the protein of the cartilage matrix, it could be very interesting to understand the consequences of a length difference in 3'UTR region on the localisation of *MATN1 *mRNA. It cannot be excluded that the length of this microsatellite marker internal to *MATN1 *gene has a functional role.

Some authors have recently suggested to proceed cautiously when interpreting the results derived from association studies for multifactorial diseases. In genome-wide association analyses, the risks that even statistically convincing evidence can occasionally lead to accept for true misleading disease pathways have been pointed out [26]. Furthermore, associations studies are based on the preliminary assumption that offspring genotypes are always formed strictly following Mendelian probabilities, but there is some evidence that transmission distortions could occur for some human genome loci [27].

Said all this, linkage analysis is a very valuable investigation tool, used also for epidemiological studies of disease aetiology as well as for clinical purposes. For instance it permits the clinical diagnosis of genetic abnormalities when the gene causing the disorder cannot be directly identified, and the presence of DNA markers can help to predict whether a foetus or a person with no symptoms carries the defective genes, which run in the family.

Although the linkage disequilibrium analysis by TDT is less efficient than standard case-control studies and uses only heterozygous parental genotypes [28], it is based on a powerful test, unaffected by stratification. The relevant indications obtained from the present study encourage to progress with the entire sequencing of *MATN1 *gene to ascertain if the 103 bp of the internal microsatellite marker plays a real functional role and is directly involved in the development of IS or weather the observed association rather relies on polymorphisms at a different level of the same gene.

## Appendix

LINKAGE A specific genetic relation between loci.

ASSOCIATION An existing relation between alleles/phenotypes.

LINKAGE DISEQUILIBRIUM The non-random association of alleles at linked loci [28].

MICROSATELLITE DNA sequence consisting of a short tandem repeat varying in number.

TDT Transmission disequilibrium test to assess linkage and linkage disequilibrium introduced by Spielman et al. (1993). The test is based on the assumption of the unequal probability of transmission of two different marker alleles from parents to affected offspring, when the marker locus and the hypothetical disease locus are linked and are in linkage disequilibrium [18].

ETDT Extended transmission disequilibrium test. It represents an extension of the TDT using logistic regression to analyse multi-allele marker loci. It was introduced by Sham and Curtis (1995) [18].
